# Effect of Chest Physiotherapy on Improving Pulmonary Function in Dealing With Congenital Heart Disease and Lung Collapse: A Case Report

**DOI:** 10.7759/cureus.33433

**Published:** 2023-01-06

**Authors:** Manali A Boob, Moli Jain, Divya M Badjate

**Affiliations:** 1 Department of Cardio-Respiratory Physiotherapy, Ravi Nair Physiotherapy College, Datta Meghe Institute of Medical Sciences, Wardha, IND; 2 Department of Physiotherapy, Ravi Nair Physiotherapy College, Datta Meghe Institute of Medical Sciences, Wardha, IND

**Keywords:** chest vibration, lung squeeze technique, prolonged slow expiration, flacc scale, patent ductus arteriosus

## Abstract

Patent ductus arteriosus (PDA) is a condition in which the ductus arteriosus fails to close after birth. We present the case of a three-month-old female infant admitted to a tertiary care center with the complaint of cough, poor feeding, and breathing difficulties. Based on the investigatory finding, she was diagnosed with PDA with pulmonary arterial hypertension (PAH), an atrial septal defect (ASD), coarctation of the aorta (COA), and pneumonia. In this patient, a left posterolateral thoracotomy was done to accomplish PDA ligation and coarctation repair. The outcomes of the infants were documented using the face, legs, activity, cry, consolability (FLACC) scale, and arterial blood gas analysis. The therapeutic aim specified an increase in oxygen saturation, enhanced total functional capacity, optimized respiration, cleared chest secretions, and normalized cardiovascular function. The effectiveness of the cardiorespiratory physiotherapy treatment regimen based on the patient's existing state of health is the focus of this case report. The outcome variable indicated that the patient's functional recovery was optimal.

## Introduction

Congenital cardiac disorders are commonly classified as cyanotic or acyanotic depending on clinical manifestations. After birth, heart structures such as the foramen ovale, ductus venosus, and ductus arteriosus are no longer necessary to sustain life [1]. The ductus arteriosus is a shunt between the pulmonary artery and the aorta that permits oxygenated blood transfer from the placenta to pass the fetal lungs and enter systemic circulation [2]. Patent ductus arteriosus (PDA) is the failure of the ductus arteriosus to close after birth, which is largely a preterm disease. The ductus arteriosus remains open in 64% of infants born at 27 to 28 weeks of gestation and in 87% of infants born at 24 weeks [2]. For hemodynamically significant PDA closure, PDA ligation, catheter intervention, or oral paracetamol may be suggested as lifesaving approaches [3]. An atrial septal defect (ASD) is a gap in the interatrial septum that allows blood to flow freely between the left and right sides of the heart. ASD is the third most prevalent kind of congenital heart defect (CHD), accounting for 30% to 40% of all CHDs [4]. Coarctation of the aorta (CoA) is a cardiac condition that causes a blockage in the aorta's circulation. The CoA is most usually observed slightly distal to the left subclavian artery, at the site where the ductus arteriosus unites with the aorta. Typically, there is medial hypertrophy with "shelf-like" tissue extending into the aortic lumen from the posterior aortic wall [5]. Pulmonary arterial hypertension (PAH) is characterized by increased pulmonary blood circulation, which leads to increased pressure in the pulmonary artery, which is often associated with cardiovascular dysfunction [6]. Bacterial pneumonia is a severe lung infection characterized by lower airway inflammation that primarily affects infants, young children, and the elderly. This disorder can be lethal if not treated immediately. Early indications and symptoms include a productive cough, a high temperature with chills, trouble breathing, tachypnea, and exhaustion [7].

## Case presentation

Here, we report a case of a three months old female child who was brought to a tertiary care center with complaints of wet cough, poor feeding, and respiratory distress. Her mother was primigravida at term gestation with oligohydramnios and delivered via lower segment cesarean section. After one month of birth, she had repeated complaints of poor weight gain, lethargy and presence of a suck-rest-suck cycle, and pneumonia and admitted to a private hospital where investigations such as a two-dimensional (2D) echo were done and she was diagnosed as a case of patent ductus arteriosus and pulmonary artery hypertension and advised surgery and referred to a tertiary care center for surgery. On admission, the patient’s parents came with the complaint of fever, cough, difficulty in breathing, and difficulty in sucking the milk so investigations were done. The chest x-ray revealed aspiration pneumonia with left-sided left lung collapse. As a result, surgery was postponed and she was admitted to the pediatric intensive care unit (PICU), where she was on ventilator support for six days and then shifted to oxygen support for 15 days mentioned in (Table 1). She underwent physiotherapeutic treatment during this time and it helped in her recovery from lung collapse. After the infection was resolved, the patient underwent surgery for PDA ligation and coarctation repair via left posterolateral thoracotomy (partial pleura over descending thoracic aortic (DTA) opened. DTA aortic arch and left subclavian artery were dissected and looped) and was advised physiotherapy post-operatively.

**Table 1:** Mode of ventilator CPAP: continuous positive airway pressure; SIMV: synchronized intermittent mandatory ventilation

Mode of ventilator	Duration
Ventilator CPAP mode	Three days (week one)
Ventilator SIMV mode	Six days (weeks one and two)
Oxygen support (4L/min) via nasal prongs	Nine days (weeks two and three)

Clinical examination

**Figure 1:**
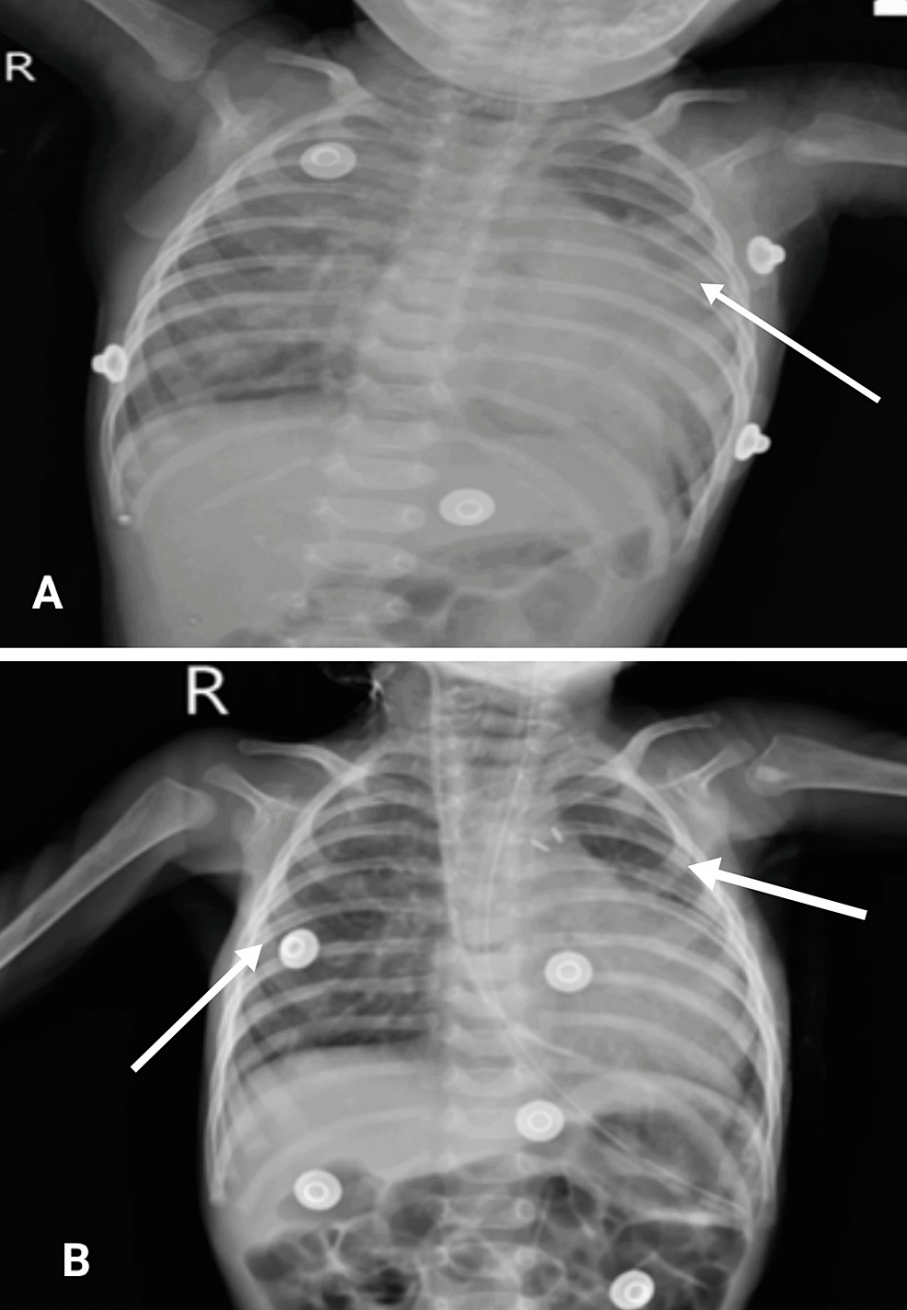
Chest radiograph (A) Pre-rehabilitation and pre-surgery chest X-ray showing left-sided lung collapse, heterogeneous opacities in the right lung field, and an obliterated cardio-phrenic angle; (B) Post-rehabilitation and post-surgery chest X-ray showing improved air entry to bilateral lung fields.

Oral consent was obtained from the patient’s parents before the beginning of the examination procedure. On inspection and observation, the patient was observed in the supine lying position. The patient was ectomorphic in her body type, conscious, cooperative, and monitored through chest leads. She was breathing through nasal prongs and feeding through a Ryle’s tube. On general examination, the patient was febrile with a pulse rate of 146 beats per minute and a respiratory rate of 46 breaths per minute, which suggests the patient was tachypneic. The patient weighed two and a half kg, had a height of 53 cm, a basal metabolic index of 8.9, a body surface area of zero point two, and a head circumference of 32 cm. On auscultation, a mid-diastolic mitral flow murmur and bilateral crepitations were present in the upper and lower zones. There was a developmental delay; the patient did not achieve the neck control milestone as she completed three months of age. A peripheral smear shows predominantly normocytic hypochromic RBCs with anisopoikilocytosis showing pencil cells and microcytes, suggesting iron deficiency anemia. Platelets are reduced on a smear. Antigen-presenting cell: 26,000 cells/mm3 as per the cell counter. Endotracheal secretion culture suggests the growth of Acinetobacter species. The chest X-ray is shown in Figures 1A and 1B, the pre-operative 2D echo, and the color Doppler study suggest left atrium/left ventricle dilation, a large nonrestrictive PDA, and left to right shunt. The left ventricle inner dimension was 3.3 cm (Z score 3.3), the left atrium was 1.6 cm (Z score 0.27), the interventricular septum was 0.6 cm (Z score 2.3), and the pulsed wave was 0.5 cm (Z score 3.0). The shelf at the isthmus; a gradient of 33 mmHg; and the coarctation of the aorta with a small Ostium Secundum-Atrial Septal Defect were observed. The timeline of the event is shown in Table 2.

**Table 2:** Treatment timeline PDA: patent ductus arteriosus; PAH: pulmonary arterial hypertension; PICU: pediatric intensive care unit

Events	Date
Diagnosed as PDA with PAH	15/12/2021
Referred to the tertiary care centre	23/02/2022
Lung infection and collapse; transferred to the PICU; and put on mechanical ventilator support.	25/02/2022
Pre-operative physiotherapy commencement (Table 3)	25/02/2022
The patient was weaned off from ventilator and put on oxygen support (4 L/min)	07/03/2022
The patient underwent PDA ligation surgery	16/03/2022
Postoperative physiotherapy begins	17/03/2022

Therapeutic interventions

In this report, the primary objective is to highlight the preoperative chest physiotherapy (Table 3) administered with continuous positive pressure for aspiration pneumonia and left lung collapse to make the patient fit for surgery, minimize the work of breathing, reduce airway resistance, and increase surge gaseous exchange, followed by postoperative physiotherapy management focused on the advanced techniques to prevent lung complications, improve breathing patterns, remove secretions, and normalize lung condition. Table 4 displays physiotherapeutic interventions.

**Table 3:** Pre-surgery physiotherapy intervention

Physiotherapeutic goals	Physiotherapeutic rehabilitation	Rehabilitation regimen
To educate the patient’s guardians regarding the condition and the operative procedure the patient is going to undergo	A counselling session was carried out for the patient’s guardians	Just prior to the surgery
To enhance the lung compliance and thoracic expansion of the patient	Passive end-expiratory pressure applied at the end of inspiration	One set of 10 repetitions was given
To promote the excursion of thoracic movement and epigastric movement	Peri-oral pressure was given	Three repetitions with a five-second hold
To optimise breathing and promote secretions' migration up from the lungs' base into the considerably larger airways	Bronchial drainage and percussion were given every two hours	Positioning with the help of the pillows was given
To improve the joint flexibility and mobility of the patient	Passive movements were given to the patient	One set of 10 repetitions was given for each joint

**Table 4:** Post-surgery physiotherapy intervention PSE: prolonged slow expiratory; LST: lung squeezing technique

Sr.	Physiotherapeutic goals	Physiotherapeutic rehabilitation	Rehabilitation regimen
1.	To make the patient's parents aware of the problem and to get their cooperation and approval for a further plan of management.	Education and counselling for caregivers regarding the patient's condition and the need for the following physiotherapeutic measures.	At the commencement of the intervention, caregivers were taught about the role of physiotherapists in the patients' care.
2.	To improve ventilation/perfusion matching	Positioning using a cushion is beneficial because a baby's lungs are not supported by the thoracic cavity, and the baby's normal resting pleural pressure is closer to atmospheric pressure than in adults, causing airway closure in more dependent zones.	Every two hours, alternate positioning to the opposite side.
3.	To remove excess phlegm secretions from smaller airways to the centre of the chest	1. Chest percussion: A percussor cup is used to percuss various portions of the chest wall based on auscultatory results. 2. Expiratory vibration: The neonatal resuscitation mask is attached to the nebulizer machine, which provides a vibratory effect when it is powered on.	Holding the percussor cup between the fingers, gently pat the infant's chest and back for two to four minutes. Vibration is given for five minutes during the expiratory phase, from the periphery to the centre of the chest.
4.	To promote clear airways and eliminate mucosal secretions and foreign particles.	Suctioning: oropharyngeal and nasopharyngeal suctioning were done as required.	To remove secretions from the central airways following chest physiotherapy.
5.	To minimise pulmonary congestion in infants suffering from pneumonia.	The PSE technique [8]: The patient is lying supine with the therapist placing one hand on the chest cavity and the other on the peritoneal cavity. At the termination of the exhalation phase, the therapist applies compressive pressure in the caudal direction from the above hand and in the cranial direction from the below hand.	The compressive force is sustained for four to five seconds following a gradual relaxation. Three sets of three compressions are applied three times a day, with a 30-second break between each compression.
6.	To restore uniform pulmonary inflation with low-frequency rhythmic thoracic compressions	The LST technique: The physiotherapist places one hand on the patient's anterior thoracic wall and the other on the posterolateral aspect, then applies sustained compression for five seconds.	Each set includes two to five thoracic compressions, three times per day. The hemithorax should be compressed for at least ten minutes, five minutes maximum on each side.
7.	To promote the establishment of suitable normal movement patterns, and as a result, noted an improved breathing pattern.	Vojta technique: The infants progressed via the stage of reflex rolling in a supine position. Slight tilting of the head in the direction where the stimulation is given. Each simulation consists of a slight amount of pressure being applied to the spine diagonally in the dorsal, medial, and cranial directions.	Every session comprises four stimuli, two given to the left side of the thorax and the other two stimuli given to the right side of the thorax. The therapy must be performed three times every day.
8.	To avoid muscle tightness and to enhance joint stability and flexibility	Passive joint mobility exercises for bilateral upper and lower extremities.	10 repetitions x one set a couple of times a day in the beginning, then gradually progressed to 10 repetitions x two sets, three times a day.

**Figure 2:**
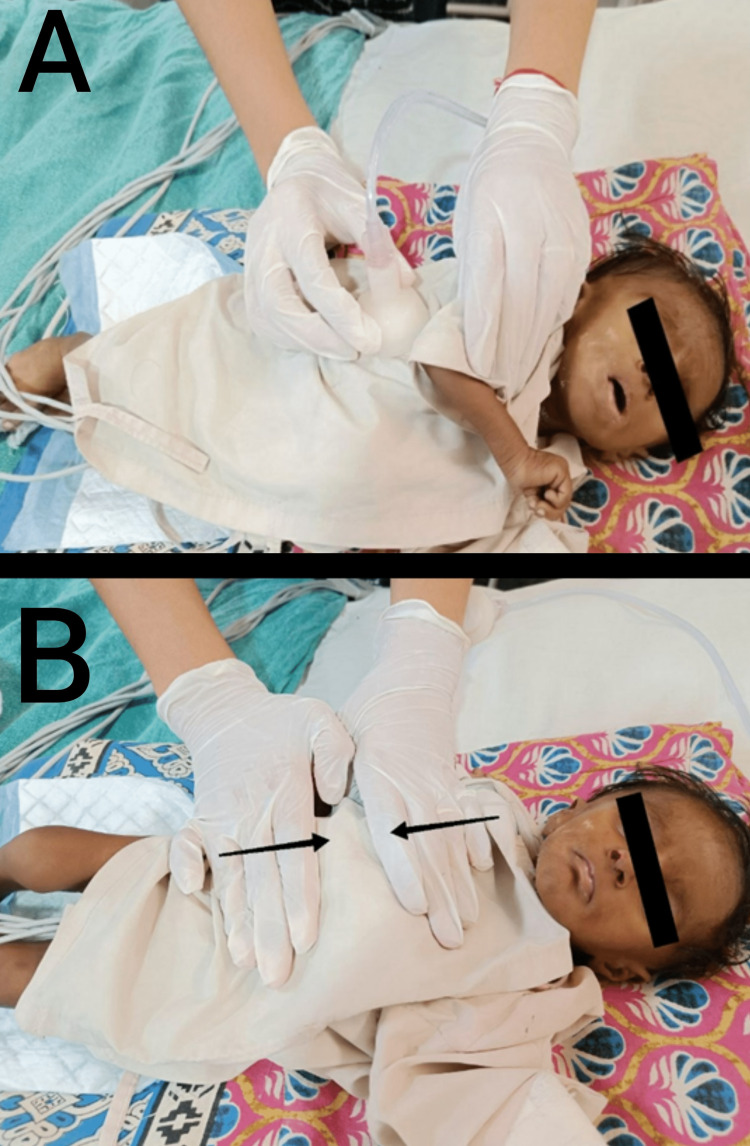
Postoperative physiotherapy intervention is shown on the patient. (A): chest vibrations with a percussor cup; (B): manual, prolonged, slow expiration technique.

Figures 2A, 2B show the post-operative physiotherapy management of a three-month-old female infant.

Outcome measures

The outcomes are shown in Table 4 and Table 5.

**Table 5:** FLACC scale interpreatation FLACC: face legs activity cry consolability

FLACC			
Components	Postoperative physiotherapy: intervention (day 1)		Postoperative physiotherapy: day of discharge
Face	1 – occasional grimace		0 – no particular expression or smile
Legs	1 – uneasy, restless, tense		0 – normal position
Activity	2 – jerking		0 – moves easily
Cry	2 - sobs		1 – occasional complaints
Consolability	2- difficult to console		0 – content, relaxed

**Table 6:** ABG analysis ABG: arterial blood gas

ABG analysis	Pre-operative physiotherapy: day 1	Pre-operative physiotherapy: last day	Postoperative physiotherapy: day 1	Post-operative physiotherapy: day of discharge
PH	6.556	7.224	6.891	7.321
PaCo_2_ (mmHg)	49.3	38.2	51.8	37.2
PaO_2_ (mmHg)	120.2	75	115.1	73.8
HCO_3_ˉ(mmol/L)	24.6	22	27.2	24
SaO_2_ (%)	83	92	90	95

## Discussion

Novel chest rehabilitation strategies, particularly those designed for infants, have been recently employed. According to the literature, the effect of PSE on breathing mechanics in infants suggests that PSE deflates the lung to increase expiratory reserve volume (ERV) and causes no modifications in peak expiratory flow (PEF), triggers sigh breaths, and reduces tidal volume, which is usually the primary mechanical feature for mucus removal [8]. Exercise is a successful therapy for CHD in emerging adults, as per earlier research. However, there is a paucity of research on how exercise affects newborns with CHD who have had a cardiac catheterization. Passive exercises and physical activity may be beneficial to infants with CHD by enhancing cardiac function and boosting bone strength [9]. The evacuation of secretions to the centre of the airway is made easier by percussion and vibration. Percussion was chosen by 74% of those physiotherapists as the preferred chest physiotherapy treatment for infants [10]. The infants developed severe acute pulmonary infectious diseases, prompting an intensive care unit stay, intubation, and ventilator support. These are all the recognised components that increase the likelihood of Acinetobacter, a multidrug-resistant bacterium that causes the spread of infection [11, 12]. The impacts of a developmental physiotherapy regimen on vital signs in preterm infants were demonstrated by the researchers. Physical therapy's advantages and hazards for specific developmental progress and for causing hemodynamic stress are significant factors in supporting intervention with preterm babies. The results of this study indicate that preterm infants responded to six developmental activities with a significant increase in mean heart rate but no significant change in mean Sao2 [13].

## Conclusions

The therapeutic purpose was to maintain tissue perfusion, promote better functional capacity, optimise breathing, completely eradicate chest secretions, and restore normal cardiovascular and respiratory function. The effectiveness of cardiorespiratory physiotherapy treatments like the prolonged slow expiratory technique, lung squeeze technique, and other pulmonary physiotherapy regimens show positive results in minimising atelectasis in the patient with pneumonia. The implication of pre-and post-operative physiotherapy strategies aids in the recovery from the pathologic condition helps avoid complications and has shown optimum functional recovery.
